# Improvement in social anxiety following a return-to-work intervention for patients with depression

**DOI:** 10.1097/MD.0000000000028845

**Published:** 2022-02-18

**Authors:** Yoko Okamoto, Rieko Takanashi, Chihiro Sutoh, Yuki Domon, Mayuko Yamada, Yoko Baba, Chiaki Aya, Naoto Yamanouchi, Hajime Sasaki, Eiji Shimizu

**Affiliations:** a Department of Cognitive Behavioral Physiology, Graduate School of Medicine, Chiba University, 1-8-1, Inohana, Chuou-ku, Chiba-shi, Chiba, Japan; b Research Center for Child Mental Development, Chiba University, 1-8-1, Inohana, Chuou-ku, Chiba-shi, Chiba, Japan; c Kokorono Kaze Chiba Clinic, Tsukamoto Chiba Third Building 9F, 2-5-15 Fujimi, Chuo-ku, Chiba-shi, Chiba, Japan; d Cognitive Behavioral Therapy Center, Chiba University Hospital, 1-8-1, Inohana, Chuou-ku, Chiba-shi, Chiba, Japan; e Department of Psychology, Faculty of Liberal Arts Teikyo University, 359 Otsuka, Hachioji, Tokyo, Japan.

**Keywords:** cognitive behavioral therapy, depression, liebowitz social anxiety scale, return to work, social anxiety

## Abstract

**Purpose:**

To retrospectively examine depression and social anxiety improvement in patients on sick leave due to depression who participated in a return-to-work intervention (RTW-I) program.

**Methods:**

Patients visited a psychiatric outpatient clinic simulating workplaces to learn recurrence prevention skills through RTW-Is, including group cognitive behavioral therapy, from April 1, 2013, to September 30, 2017. The Beck Depression Inventory-Second Edition (BDI-II), Social Adaptation Self-Evaluation Scale (SASS), and Liebowitz Social Anxiety Scale (LSAS) scores of 112 patients were analyzed before and after the intervention program. Missing postprogram data were substituted using the last observation carried forward scores. Next, 45 patients who responded to the work continuity survey 1 year after RTW-I were categorized into Group A (patients who continued working: 37) and Group B (those who did not continue: 8).

**Results:**

The mean BDI-II scores significantly decreased from preintervention 19.4 to postintervention 7.9 (*t* = 13.303, *P* < .001). The mean SASS scores significantly increased from preintervention 31.9 to postintervention 36.0 (*t* = −5.953, *P* < .001). The mean LSAS scores significantly decreased from preintervention 54.7 to postintervention 37.0 (*t* = 8.682, *P* < .001), and all scores demonstrated an improvement. Patients who continued working showed improved depressive and social anxiety symptoms. The BDI-II and SASS scores showed no significant differences between the groups, but the postintervention LSAS scores were significantly different (*P* = .041). LSAS score changes: Group A = −26.2; Group B = −9.8; estimated difference: −17.920, 95% CI: −32.181 to −3.659, *P* = .015.

**Conclusions:**

The RTW-I program improved depressive and social anxiety symptoms. Patients with improved scores continued working for 1 year after the intervention.

**Trial registration:** This trial was retrospectively registered with the UMIN Clinical Trial Registry (UMIN-CTR) (ID: UMIN000037662) on August 10, 2019.

## 1 Introduction

### 1.1 Economic losses due to sick leave taken by patients with depression

Depression is a common mental disorder (CMD) with a high lifetime prevalence of 6.2% among local residents in Japan.^[1]^ In recent years, the need for interventions for subthreshold depression that does not meet the diagnostic criteria for major depression has become apparent, as subthreshold depression is a risk factor for major depressive disorder and decline in labor productivity.^[^2^,^^3^^]^ Research on the financial costs of depression has shown that absenteeism (loss of productivity due to absence from work) or presenteeism (loss of productivity while working) leads to significant economic losses.^[4]^ Moreover, the recurrence rate for depression is as high as 60%. Patients with depression who experience a relapse after returning to work may remain absent for several months and struggle to return to work after repeated absences, disrupting the continuity of employment.^[5]^

### 1.2 Response to mental health measures in Japan

In 2012, the Japanese Ministry of Health, Labor and Welfare revised and recommended the “Guidance for supporting return-to-work for workers who have taken sick leave due to mental health problems” as a manual in the workplace.^[6]^ Additionally, since December 2015, “The Stress Check Program,” based on the Industrial Safety and Health Law amendment, Japan, has been employed in response to mental health issues in the workplace. Japanese companies have also launched various initiatives to promote mental health. However, given the increasing number of workers affected by mental health problems and the difficulties associated with promoting mental health in the workplace, employees seeking support participate in return-to-work support intervention programs (RTW-Is) at psychiatric clinics.^[7]^ RTW-Is aim to assist patients’ recovery from symptoms of mental health problems such as depression, aid in preparations for returning to work, and enable patients to fulfill their roles at work on returning to the workplace.

### 1.3 Global efforts to support return to work

The social costs associated with the loss of productivity resulting from absenteeism due to mental health problems have led to global efforts to address mental health problems at the workplace.

Since 2000, the Netherlands has been a leader in supporting patients returning to work, having developed guidelines for general and occupational health physicians to support the reinstatement of workers after sick leave due to mental disorders.^[8]^ These guidelines aim to limit the economic effects of labor loss due to sick leave and include steps in diagnosis, intervention, and prevention of relapse, enabling individuals with mental health problems to solve issues at work using cognitive behavioral therapy (CBT).^[8]^ Numerous randomized controlled trial studies support the use of these guidelines.^[^9^–^11^]^

In the Netherlands, occupational physicians are actively involved in return-to-work interventions, as shown in a randomized controlled trial. In a study of individuals with mental health problems due to stress, a group that underwent step-by-step stress management based on CBT conducted by occupational health physicians and a group who underwent typical care were compared; the intervention group returned to work earlier and showed a lower frequency of relapse.^[11]^

In a meta-analysis of 48 experimental studies investigating the effects of interventions related to reducing occupational stress, CBT was found to be moderately effective and more effective than relaxation and other interventions.^[10]^

### 1.4 Efforts to support return to work in Japan

In 1997, Akiyama incorporated occupational therapy into a rehabilitation program to support return to work for employees on leave due to mental disorders, marking the start of this kind of support in Japan. Modern RTW-Is are offered at medical facilities, Vocation Support Centers for Persons with Disabilities, and nongovernment institutions that have Employee Assistance Programmes, which provide rehabilitation for workers on leave due to mental disorders to facilitate their return to work.^[12]^

Thus far, few clinical studies have investigated the efficacy of RTW-Is. One exception is a study by Ohki et al,^[13]^ who examined 12 domestic effectiveness studies in the literature and assessed the short- (recovery from clinical symptoms and achievement of reinstatement) and long- (work continuity and recurrence of mental health problems after returning to work) term efficacies of an RTW-I. Although many studies have demonstrated the usefulness of the program, most of them were single-arm studies, and only one was a randomized controlled trial, rendering the accumulation of studies with a strong scientific basis a challenge.

### 1.5 CBT intervention

CBT is widely applied to CMDs, such as depression and social anxiety, and the current RTW-I includes group CBT for depression. The National Institute of Clinical Excellence guidelines in Great Britain^[14]^ recommend individual CBT as the first-line treatment for depression. In addition, a randomized controlled trial conducted by Clark et al^[15]^ showed that CBT was more effective than pharmacotherapy in treating social anxiety. Standard psychotherapy treatment programs in medical institutions differ in content depending on institutional protocols; however, they frequently include CBT and commonly regard interpersonal relationships after returning to work as the main target area. Many medical institutions favor group over individual programs.^[16]^

### 1.6 Feasibility of evaluation of return-to-work initiatives for social anxiety disorder

Social anxiety disorder (SAD) is a CMD and is prone to chronicity. An epidemiological survey on mental disorders in the United States found that 10% to 15% of the population showed an onset of SAD in their mid-teens to early twenties, with an average morbidity duration of 10 to 20 years or more.^[17]^ SAD is linked to extreme anxiety around public appearances or attending group events. As a result, patients with SAD struggle to participate in social situations, such as attending work or school. However, many patients with SAD are unaware of their disability despite experiencing serious problems in their interpersonal relationships at work. In fact, most individuals experience symptoms of SAD for several years before receiving treatment. They commonly attribute their difficulties to deficiencies in their abilities or personalities, which renders them prone to mental breakdowns.^[18]^

In Japan, school refusal, social withdrawal, and “Not in Education, Employment, or Training” statistics for SAD patients who have difficulty taking part in social activities estimate labor losses due to absences to be around 1.5 trillion yen per year.^[19]^ The incidence of SAD may be underdiagnosed, as patients with SAD seldom mention their symptoms (e.g., tension in public situations) during medical consultations.

Therefore, it is important to properly evaluate the social anxiety levels of RTW-I participants who are on sick leave due to depression so that their social anxiety and depression are properly diagnosed and treated to ensure a successful return to work. While many clinical studies have examined the improvement in depressive symptoms among workers,^[13]^ few studies have examined improvements in social anxiety symptoms. Therefore, the aim of this study was to assess the changes in social anxiety symptoms after an RTW-I.

### 1.7 Aims of the study

We retrospectively analyzed questionnaire data and medical records from outpatients who were on sick leave due to mental disorders, such as depression, and participated in the RTW-I. Furthermore, we investigated whether the improvement in social anxiety symptoms was related to the success or failure of reinstatement and continued work after reinstatement. We aimed to evaluate the usefulness of the program by examining the changes in social anxiety symptoms.

## 2 Methods

### 2.1 Ethical statement

Ethical approval was obtained from the Ethical Review Committee of Chiba University Graduate School of Medicine (No. 2522; approval date: 11/30/16). Obtaining informed consent to participate in the study was not applicable in this case. During the opt-out consent process, patients were notified of the use of their clinical information for the research. The notice was posted on the website of Chiba University and a bulletin board in the Rework Day Care Outpatient Clinic hospital. None of the patients refused permission for the use of their medical information in this study.

### 2.2 Participants

We retrospectively analyzed the data for patients who participated in the RTW-I at Kokorono Kaze Chiba Clinic, which provides specialized depression reinstatement support treatment programs to outpatients.

Inclusion criteria were as follows: patients enrolled in the RTW-I from April 1, 2013, to September 30, 2017; patients who did not refuse the use of personal medical information in the study; patients aged >20 years; and availability of preintervention data from the Beck Depression Inventory-Second Edition (BDI-II), Social Adaptation Self-Evaluation Scale (SASS), and Liebowitz Social Anxiety Scale (LSAS) in the full analysis set (FAS). Missing postprogram data were substituted using the last observation carried forward scores. Patients still participating in RTW-Is were excluded.

### 2.3 Design

Of the 186 participants enrolled in the RTW-I between April 1, 2013, and September 30, 2017, 112 were included in the study. A total of 74 participants were excluded, including 31 patients still participating in RTW-Is and 43 without complete preintervention data from the BDI-II, SASS, and LSAS (the LSAS survey was added in July 2014). BDI-II, SASS, and LSAS psychological and work functioning assessments for 112 patients were conducted before the return-to-work program (“preintervention”) and post the return-to-work program (“postintervention”). Next, we divided the 45 patients who responded to the work continuity follow-up survey 1 year after the program completion into 2 groups: Group A (37 patients who continued to work) and Group B (8 patients who did not continue to work).

#### 2.3.1 Beck depression inventory-second edition

The Japanese version of the BDI-II,^[20]^ a self-administered questionnaire that assesses the severity of depressive symptoms, was used in this study.^[^21^,^^22^^]^

This scale comprises of 21 items that are rated on a scale of 0–3. The maximum score is 84, with a higher score indicating greater severity of depressive symptoms: 1 to 10, normal range; 11 to 16, mildly depressed state; 17 to 20, boundary region; 21 to 30, medium depression; 31 to 40, severe depression; and ≥41, extreme depression. The boundary region indicates borderline depression, and a score of >17 requires specialist treatment.

#### 2.3.2 Social adaptation self-evaluation scale

The Japanese version of the SASS,^[23]^ a self-assessment scale that evaluates the degree of recovery of social adaptive ability in depressed patients, was used in this study.^[24]^ This scale consists of 20 items that evaluate social adaptative ability, including interest in work and housework and social interactions with family members and other individuals. The maximum score is 60, and higher scores represent better social adaptation. Goto et al^[24]^ reported a mean score of 36.5 ± 5.7 in healthy Japanese individuals, whereas Sagata et al^[25]^ reported a mean score of 28.8 ± 6.2 in patients with depression. Assessing the state of recovery of subjective social function in patients with depression is important in determining the timing of reinstatement to work.

#### 2.3.3 Liebowitz social anxiety scale

The Japanese version of the LSAS,^[26]^ a widely used symptom rating scale for SAD, was used in this study^[27]^ to evaluate the degree of “fear/anxiety” and “avoidance” on a scale of 0 to 3 for 24 action status/social situations. The total score indicates the severity of social anxiety symptoms, with higher scores reflecting greater severity. A score of 30 indicates the boundary region, 31 to 49 indicates mild anxiety, 50 to 70 indicates medium anxiety, 80 to 90 indicates more pronounced anxiety causing disability in daily life, and ≥95 indicates severe anxiety.^[27]^

### 2.4 RTW-I provided at Kokorono Kaze Chiba clinic

The RTW-I program started at the Kokorono Kaze Chiba Clinic in April 2013. Its purpose was to facilitate the return to work of patients on sick leave due to mental disorders such as depression by enhancing patients’ readiness for reinstatement and supporting a graded return to work.

Patients visited the clinic 5 days per week for 6.5 hours/day, simulating routine employment attendance, and participated in daily rehabilitation programs to acquire skills to assist in the prevention of recurrence of mental disorders and reduce the need for future sick leave. This involved relaxation, light physical exercise, creative activities, group counseling, social skills training, learning self-control by behavior activation and CBT, coping with stress, interpersonal communication skills, and group CBT. Approximately 4 to 6 months are necessary for patients to acquire these skills.

The RTW-I staff comprised a multi-professional team involving psychiatrists, nurses, clinical psychologists, psychiatric social workers, and occupational therapists.

### 2.5 Data analyses

Paired *t*-tests were performed to examine changes in the pre and postintervention BDI-II, SASS, and LSAS scores. In addition, correlation analyses were performed to assess the relationship between changes in scores using Pearson's correlation coefficient. We employed the Chi-Squared or Fisher exact test for categorical variables and Student *t* test for the other variables.

We divided the patients who responded to the work continuity follow-up survey 1 year after the program completion into 2 groups, following which their pre and postintervention BDI-II, SASS, and LSAS scores were analyzed by unpaired *t*-tests and Fisher exact test. Differences were considered significant if *P* < .05, with two-tailed testing. As a sensitivity analysis, differences in changes in BDI-II, SASS, and LSAS scores between groups were evaluated using analysis of covariance adjusted for preintervention scores, sex, and age as covariates. All of the data were analyzed using IBM SPSS Statistics for Mac, Version 25.0 (IBM, Armonk, New York).

## 3 Results

A total of 186 patients participated in the program, 112 of whom were eligible. In total, 74 participants were excluded, including 31 patients still participating in RTW-Is and 43 without complete preintervention data from the BDI-II, SASS, and LSAS (the LSAS survey was added in July 2014). In performing the intention-to-treat analysis, the data from 112 patients were analyzed as a FAS, with preintervention BDI-II, SASS, and LSAS scores. Any missing postintervention data were substituted using the last observation carried forward scores. Complete pre and postintervention data sets existed for 85 of the 112 patients and were analyzed as per the protocol set.

### 3.1 Participant characteristics and BDI-II, SASS, and LSAS scores using the FAS

The participant characteristics in the FAS are shown in Table 1. The mean clinical visit duration of the RTW-I was 276.5 days (SD = 144.4). The mean number of instances of sick leave was 1.9 (SD = 1.1). In addition, 75.9% of the patients were living with their family, whereas 24.1% of the patients lived alone.

**Table 1:**
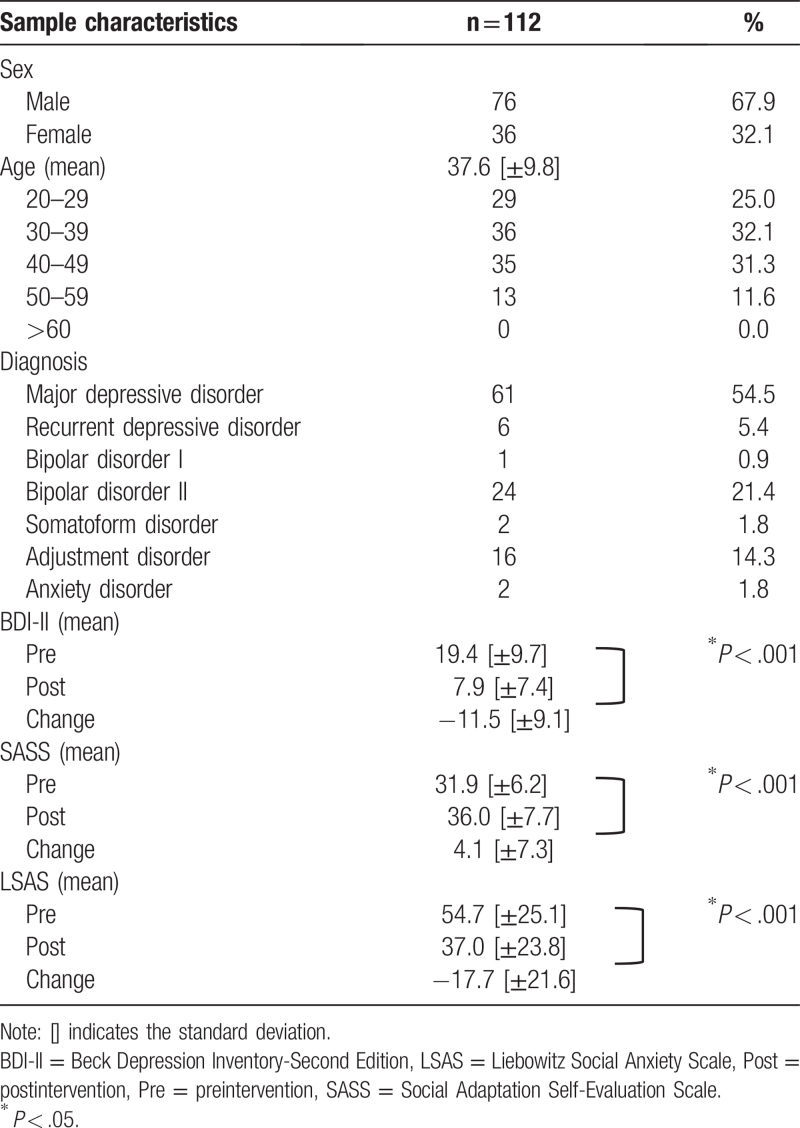
Participant characteristics in the full analysis set.

Most of the participants worked in the civil services sector (26.8%). Other sectors were represented as follows: manufacturing, 17.9%; communication business, 10.7%; service industry, 10.7%; finance and insurance, 8.9%; wholesale and retail, 0.9%; and others (academic research, medical and welfare, and electricity and gas), 24.1%. Furthermore, 70% of the participants were technical specialists (47.3%) or office workers (26.8%). General employees (71.4%) and section or team heads (20.5%) accounted for 90% of the job positions in this cohort.

### 3.2 Pre and postintervention changes in BDI-II, SASS, and LSAS in the FAS

The mean BDI-II scores significantly decreased from 19.4 (SD = 9.7) preintervention to 7.9 (SD = 7.4) postintervention (*t* = 13.303, *P* < .001), indicating that patients experienced fewer symptoms of depression. Specifically, symptoms of depression improved from the boundary region (17–20 points) to the normal range (0–10 points).

The mean SASS scores significantly increased from 31.9 (SD = 6.2) preintervention to 36.0 (SD = 7.7) postintervention (*t* = −5.953, *P* < .001), indicating that patients’ self-evaluated social adaptive ability had improved. Specifically, the self-rating of social adjustment improved to near the mean score of 36.5 ± 5.7 in healthy Japanese individuals.

The mean LSAS scores significantly decreased from 54.7 (SD = 25.1) preintervention to 37.0 (SD = 23.8) postintervention (*t* = 8.682, *P* < .001), indicating a reduction in social anxiety (Table 1). Specifically, social anxiety symptoms improved from medium anxiety (50–70 points) to the boundary region (about 30 points).

### 3.3 Correlation between LSAS changes and BDI-II and SASS changes

We found a significant positive correlation (*r* = 0.415, *P* < .001) between the LSAS and BDI-II scores, and a significant negative correlation (*r* = −0.465, *P* < .001) between the LSAS and SASS scores (Table 2).

**Table 2:** Correlation between LSAS changes and BDI-II and SASS changes in the FAS.

	BDI-II change	SASS change
LSAS change	0.415^∗∗^	−0.465^∗∗^

BDI-II = Beck Depression Inventory-Second Edition, LSAS = Liebowitz Social Anxiety Scale, SASS = Social Adaptation Self-Evaluation Scale.

∗∗ The correlation coefficient was considered significant if *P* < .01 (two-tailed).

### 3.4 Relationship between improvements in SAD symptoms and continuation of work at 1 year postintervention

Table 3 summarizes the differences in pre and postintervention BDI-II, SASS, and LSAS scores between the 2 groups (Group A, n = 37: those who continued to work; Group B, n = 8: those who did not continue to work) as revealed by unpaired *t*-tests.

**Table 3:**
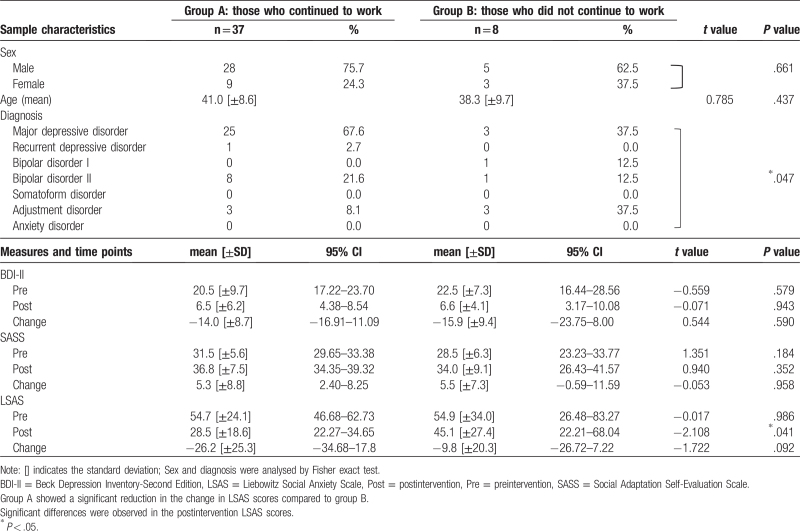
Relationship between improvements in social anxiety disorder symptoms and continuation of work at 1 year postintervention in the FAS.

The analysis revealed no significant differences in the BDI-II and SASS scores between Group A and Group B, but there were significant differences in the postintervention scores of the LSAS (*P* = .041). In addition, in Group A, the change in the LSAS score decreased by 26.2 (SD = 25.3), whereas the change in the LSAS score decreased by 9.8 (SD = 20.3) in Group B. Thus, Group A had a significant reduction in LSAS scores compared with Group B. These results indicate that patients with significantly reduced social anxiety symptoms after the intervention program were better able to continue to work 1 year after the intervention.

Differences in the changes between groups were analyzed by analysis of covariance adjusted for preintervention scores, sex, and age as covariates (Table 4). The amount of change in LSAS was significantly greater in Group A than in Group B (estimated difference: −17.920, 95% CI: from −32.181 to −3.659; *P* = .015).

**Table 4:** Changes in BDI-II, SASS, and LSAS scores and estimated differences in the FAS.

	Group A: n = 37 those who continued to work	Group B: n = 8 those who did not continue to work			
Measures	Mean [±SD]	Mean [±SD]	Estimated difference	95% CI	*P* value
BDI-II change	−14.0 [±8.7]	−15.9 [±9.4]	−0.241	−4.552 to 4.069	.910
SASS change	5.3 [±8.8]	5.5 [±7.3]	−3.423	−11.002 to 4.156	.366
LSAS change	−26.2 [±25.3]	−9.8 [±20.3]	−17.920	−32.181 to −3.659	^∗^.015

Note: [] indicates the standard deviation. Differences in changes between groups were analyzed by ANCOVA adjusted for preintervention scores, sex, and age as covariates. BDI-II = Beck Depression Inventory-Second Edition, LSAS = Liebowitz Social Anxiety Scale, SASS = Social Adaptation Self-Evaluation Scale.

∗ *P* < .05.

## 4 Discussion

The present study examined improvements in depressive and social anxiety symptoms in patients who returned to work after participating in an RTW-I in Japan. The results suggest that the RTW-I programs improved not only depressive symptoms but also social anxiety symptoms after intervention. Patients who continued to work for 1 year after the intervention showed greater improvement in social anxiety symptoms after the intervention than those who did not.

This study highlights the important role that medical facilities play in assisting patients to follow return-to-work guidelines and preventing recurrent sick leave.^[28]^ Further studies with larger cohorts are required to confirm our findings that individuals with fewer symptoms of depression and social anxiety, and better social adaptation can maintain stable work continuity.

Notably, only 1.8% of the participants in this study had a primary diagnosis of an anxiety disorder. This can be ascribed to the characteristic symptoms of social anxiety that cause these patients to misattribute the difficulties they experience in their social interactions to some deficiencies in their own personalities and capabilities. Hence, they seldom mention their symptoms during medical consultations. Therefore, the condition is frequently undiagnosed, making it essential to improve diagnostic approaches to social anxiety and raise awareness regarding social anxiety.

The lifetime prevalence of SAD in the United States and Japan is reported to be 12% and 1.8%, respectively. The frequency of diagnosis of patients with SAD in Japan tends to be lower than that in other countries, and many patients are undiagnosed.^[29]^ The onset of SAD has also been reported to precede the onset of depression. If left untreated, SAD may evolve into major depression and other mental health problems. Therefore, it is important to identify the presence of SAD in patients with depression.

A recent Dutch study examined the Return-to-Work Self-Efficacy (RTW-SE) trajectories in employees with mental health problems using the RTW-SE scale.^[30]^ They reported that the group with less favorable trajectories was characterized by higher age, a higher prevalence of anxiety disorder, and lower RTW rates.^[31]^ The findings support our research on the importance of alleviating anxiety disorders to improve return to work, and, at the same time, the report shows interest in focusing on RTW-SE in the RTW-I process.

A Norwegian study evaluated the validity of the RTW-SE among patients receiving work-focused therapy (N = 626) for common mental disorders such as depression and anxiety disorders, the results of which supported the validity of the instrument.^[32]^ Previous studies have examined the relationship between anxiety disorder symptomatology and low self-efficacy.^[^33^,^^34^^]^ Our study used the BDI, SASS, and LSAS to examine improvements in depression and social anxiety symptoms, and it would be interesting for future research to use the RTW-SE questionnaire to investigate the relationship between improvements in social anxiety symptoms and RTW-SE.

Kimura et al^[35]^ assessed life events associated with treatment-refractory depression in a refractory group (n = 31) and remitted group (n = 31) using the Impact of Event Scale-Revised (IES-R), a rating scale for post-traumatic stress disorder. The mean ± SD score of the IES-R in patients with treatment-refractory depression (46.7 [15.1]) was significantly higher than that in remitted (10.3 [9.9]) patients with major depressive disorder. They reported that studies demonstrated that patients with treatment-refractory depression perceive their onset-related life events as serious psychological distress symptoms.

Given that half a million Canadians a week are unable to work due to psychological problems, the Mental Health Commission of Canada has called for action to address psychological injury and harassment at the workplace. Australia has also sought to address work-related psychological injury at the workplace, as psychological injuries at the workplace can be a factor in depression, anxiety disorder, and post-traumatic stress disorder. In Japan, a law to prevent power harassment in the workplace was enacted on June 1, 2020. From a clinical perspective, social anxiety symptoms upon return to work may be related to interpersonal fear of perpetrators of power harassment and bullying in the workplace. In devising future RTW-Is, it is necessary to devise a program that focuses not only on reducing symptoms and improving return to work and job continuity, but also on reducing stress in the workplace.

Interpersonal relationships after returning to work are considered primary targets of RTW-Is for depression at medical institutions.^[16]^ However, few studies have examined social anxiety symptoms. Therefore, our findings regarding the improvement in social anxiety symptoms in addition to depression post-RTW-I are especially significant; particularly because besides recovery from depressive symptoms, improved self-evaluation of social adaptation and recovery of social functioning^[36]^ are essential for returning to work, where, apart from occupational performance, interpersonal skills (i.e., social activities and interactions with other people) are required.

Previous studies have demonstrated that work-focused CBT accelerates functional recovery and time to RTW compared to regular CBT.^[37]^ Ito et al^[38]^ conducted a work-focused cognitive-behavioral group therapy for 22 individuals on leave due to depression. They evaluated depression and anxiety using the Kessler-6 scale, whereas our study evaluated depression and social anxiety using the BDI-II and LSAS, respectively. They found that after the intervention, depression, anxiety, social adjustment, and difficulty in returning to work (interpersonal aspects after returning to work and cognitive functions necessary for work) had improved significantly, suggesting the effectiveness of the program. It was reported that the participants continued working after 3 months of returning to work. However, no long-term evaluation was conducted.

RTW-I participants should therefore be fully evaluated for social anxiety, measures should be taken to overcome social anxiety symptoms during interpersonal interactions, and adjustments to the workplace environment should be made to achieve adequate work performance and work continuity.

Clinical studies assessing how improvements in social anxiety relate to other symptoms, as well as reinstatement and work continuity, are required to enhance the efficacy of future RTW-Is. These RTW-Is should also attempt to simulate the various social interactions within the workplace as accurately as possible.

Our study has some limitations. Two previous studies^[^39^,^^40^^]^ have investigated return-to-work interventions using CBT, both of which included larger sample sizes than our study, which retrospectively analyzed data from only 112 patients from a single medical institution. This small sample size limited our ability to fully investigate the relationship between the decrease in the LSAS scores and work continuity after 1 year.

Furthermore, a 1 year follow-up survey was conducted using the physical mail system. The response rate of 45 respondents (40.2%) was low, and the small sample size limited our ability to fully investigate the relationship between the decrease in LSAS scores and work continuity after 1 year. More sophisticated assessments could be conducted using web-based methods, which would yield prompt responses and higher follow-up rates.^[41]^ Igarashi et al^[41]^ used a web-based system to ensure a high follow-up rate of 210 (97.7%) in a prognostic survey of users of a return-to-work support program for 2 years after their return to work. They reported that the system enabled realistic reporting with a work rate of 89.5% for approximately 2 years. However, caution is required because, as Kaldo et al^[39]^ pointed out, the usage rate of Internet based cognitive behavioral therapy work-related modules is low. More importantly, as the number of patients to be followed up 1 year after returning to work will increase in the future, a prospective study with a larger cohort would be more representative.

## 5 Conclusions

Overall, our data suggest that RTW-Is improved social anxiety and depression symptoms in patients on sick leave due to mental disorders such as depression.

These results are important for the reintegration of workers with mental health problems into the workforce, indicating that suitable RTW-I programs will enhance work continuity and reduce economic losses incurred due to long/recurrent periods of sick leave. However, it is necessary to conduct further clinical research on the impact of social anxiety symptoms on return to work, focusing on reinstatement to work and long-term work continuity after reinstatement.

## Acknowledgments

We thank the participants of this research for their valuable contributions.

## Author contributions

**Conceptualization:** Yoko Okamoto, Rieko Takanashi, Chihiro Sutoh, Eiji Shimizu.

**Formal analysis:** Yoko Okamoto, Chihiro Sutoh, Eiji Shimizu.

**Investigation:** Rieko Takanashi, Yuki Domon, Mayuko Yamada, Yoko Baba, Chiaki Aya.

**Supervision of the investigation:** Naoto Yamanouchi, Hajime Sasaki.

**Project administration:** Eiji Shimizu.

**Supervision:** Eiji Shimizu.

**Writing – original draft:** Yoko Okamoto.

**Writing – review & editing:** Eiji Shimizu.
